# Molecular Mechanisms in Lysosomal Storage Diseases: From Pathogenesis to Therapeutic Strategies

**DOI:** 10.3390/biomedicines10040922

**Published:** 2022-04-17

**Authors:** Valeria De Pasquale, Melania Scarcella, Luigi Michele Pavone

**Affiliations:** 1 Department of Veterinary Medicine and Animal Productions, University of Naples Federico II, Via F. Delpino 1, 80127 Naples, Italy; 2 Department of Molecular Medicine and Medical Biotechnology, University of Naples Federico II, Via S. Pansini 5, 80131 Naples, Italy; melania.scarcella@unina.it

Lysosomal storage diseases (LSDs) are a group of metabolic diseases caused by inborn mutations of lysosomal enzymes, which lead to lysosome substrate accumulation in various cell types. As a result, a complex variety of pathogenic cascades are triggered, resulting in a clinical profile characterized by multisystemic involvement [1]. Indeed, LSDs are featured by a broad spectrum of clinical signs depending on both substrate and site of accumulation [2]. Among common clinical manifestations, nervous system (NS) implication is one of the most frequent signs and is shared among almost all types of LSDs [2].

Although the biochemical nature of LSDs was first revealed in 1934 [3], it is only over the last two decades that a series of studies have contributed to the understanding of the pathogenic mechanisms of such diseases [4,5,6,7,8,9,10]. These studies have disclosed the important role of the altered autophagy flux [4] and mitochondrial function [5], which, along with the stimulation of inflammatory response and immune abnormalities [6], appear to represent a common feature of many LSDs. In addition, the interplay between the accumulation/mislocalization of substrates and the alteration of many signaling pathways is also gaining attention in the context of LSDs [7,8,9,10]. However, despite advances that have been made in the treatment of these diseases, current therapies are available for only a subset of them, most of which only aim to ameliorate disease symptoms [11,12,13,14,15,16,17].

This Special Issue provides a comprehensive view of the molecular aspects of various LSDs. Our goal is to collect state-of-the-art research on LSDs and their pathomechanisms, with a focus on recent discoveries that have been made on the NS involvement, such as the association between LSDs and neurodegenerative disorders, or the use of neurological LSDs models for pathogenesis investigation and drug discovery.

Clarke et al. provided interesting insights on the interplay between LSDs and protein aggregation in neurodegenerative disorders. More specifically, they analyzed seven LSDs (Gaucher, Fabry, Sandhoff, Niemann–Pick A, Hurler, Pompe, and Niemann–Pick C) murine models and found evidence of neuroinflammation and α-synuclein, both reminiscent of α-synucleinopathies such as Parkinson’s disease [18].

Moreover, the role of lysosomal and endosomal dysfunction in the pathophysiology of neurodegenerative disorders is well known. Bécot et al. provided a review that focuses on the association between endosomal system impairment and the generation of amyloid plaques in Alzheimer’s disease (AD). They highlighted how AD-related dysfunction of the endosomal system, which diverts from a degradative to a secretory function, can promote the formation of exosome and exocytosis of pathogenic amyloid species [19].

Another association between neurodegenerative disorders and lysosomal dysfunction was reported by Bicchi et al. They reported lysosomal engulfment and autophagy impairment in primary fibroblast of transgenic mice expressing a mutant form of superoxide dismutase 1 (SOD1), a gene frequently mutated in amyotrophic lateral sclerosis type-1 (ALS1) [20].

Although NS involvement is the most severe consequence of the majority of LSDs, the impairment of other organs and tissues is very common. For instance, it is estimated that 60–90% of mucopolysaccharidosis (MPS) patients, a type of LSD characterized by glycosaminoglycans (GAGs) accumulation, suffer from cardiac valve dysfunctions [21,22]. Moreover, deposition of GAGs can also occur in the vascular smooth muscle and in cardiac muscle cells. As a result, MPS is frequently associated with heart diseases such as myocardium fibrosis and coronary heart disease [23,24,25].

Additionally, a large screening study performed on kidney transplantation recipients highlighted the importance of the accurate and early diagnosis of the Fabry disease, an LSD caused by mutations in the enzyme alpha-galactosidase A (GLA), with its most life-threatening clinical sign represented by end-stage kidney failure [26].

During the last few decades, large efforts have been made to set up reproducible models that finely replicated the clinical features of LSDs patients. To this end, a widely used model organism is represented by Drosophila melanogaster: as reviewed by Rigon et al., given the evolutionary conservation of genes and pathways involved in several LSDs pathogenesis between the fruit fly and Homo sapiens, it can be used both to study molecular mechanisms underlying LSDs pathology and as a tool to new drugs and treatments discovery [27].

More recently, induced pluripotent stem cells (iPSCs) have been proven to be effective emerging tools for the study of some types of LSDs—such as metachromatic leukodystrophy (MLD) [28]. Nevertheless, factors such as the difficulty to yield somatic cells from patients, high cost, and uncertain physiological fidelity can significantly limit their employment [29]. In an attempt to overcome this limitation, Esmail et al., leveraging a deep machine-learning platform, developed artificially induced whole-brain organoids (aiWBO) as a model of MLD. aiWBO accurately simulated the phenotype of both wild-type and MLD nervous system cells. Importantly, the same research group demonstrated that such a model could also predict the efficacy of combined drug treatment that ameliorate the MLD disease profiles, rendering it eligible for new therapies discovery [30].

Altogether, the articles of this Special Issue contribute to enhancing the knowledge in the field of LSDs, with a focus on the nervous system involvement. In particular, the cited articles provide new insights on pathological mechanisms, setups of new study models, and the discovery of new therapies related to this group of metabolic diseases.
